# Reaction kinetics, mechanical characteristics, and microstructure of steel slag-cement binder modified with graphene oxide

**DOI:** 10.1039/d3ra00257h

**Published:** 2023-05-09

**Authors:** Qidong Wang, Xudong Wang, Hongxin Liu

**Affiliations:** a Yuanpei College, Shaoxing University Shaoxing 312000 China ppag2525ee0dbfe5@sohu.com@e-mail.com +86-13754392525

## Abstract

Graphene oxide (GO) was utilized as an additive to encourage the development of early strength in order to improve steel slag cement's low early strength. This work investigates the compressive strength and setting time of cement paste. The hydration process and its products were explored using hydration heat, low-field NMR, and XRD; in addition, the internal microstructure of the cement was analyzed utilizing MIP, SEM-EDS, and nanoindentation testing technologies. The results demonstrated that the inclusion of SS retarded cement's hydration, leading to a degradation of compressive strength and microstructure. However, the addition of GO was able to accelerate the hydration of steel slag cement, giving rise to a reduction in total porosity, a strengthening of the microstructure, and an improvement in compressive strength, particularly the material developing trends at the early stage. Due to its nucleation and filling capabilities, GO increases the total amount of C–S–H gels present in the matrix, specifically vast amounts of C–S–H gels with high density. It has been established that the addition of GO is capable of greatly enhancing steel slag cement's compressive strength.

## Abstract 2

Graphene oxide (GO) was utilized as an additive to encourage the development of early strength in order to improve steel slag cement's low early strength.

## Graphical abstract

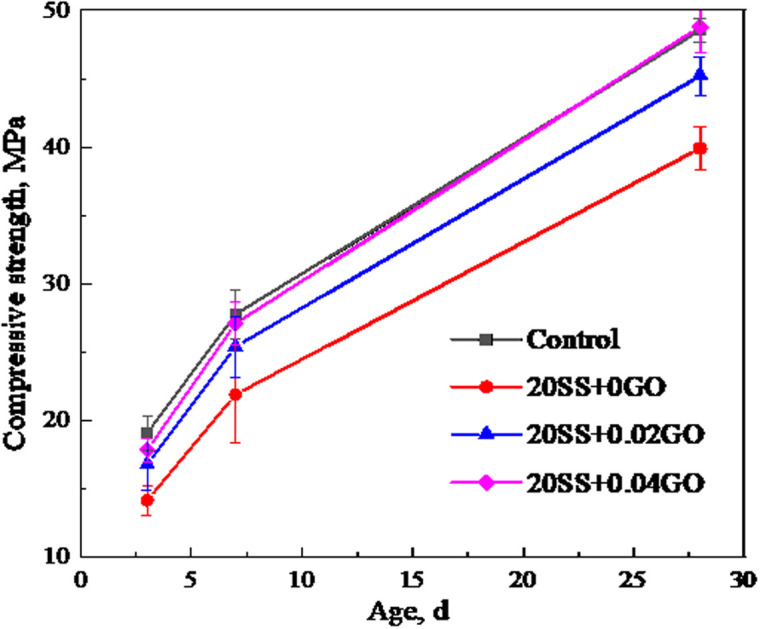

## 1 Introduction

Steel slag powder (SSP) is a common industrial waste derived from iron and steel works. According to reports, the output of steel slag accounts for 15–20% of China's crude steel output, and produces about 80 million tons per year.^1,2^ A mass of steel slag is landfilled, occupying land resources, even causing serious impact on water resources and the environment, indirectly endangering people's health.^3^ In developed countries, such as the United States, Australia, Japan, *etc.*, the utilization rate of steel slag is as high as 85–98%. However, the utilization rate of steel slag in China is only 22%.^4^ Thus, how to effectively utilize steel slag is imminent.

Currently, the resource utilization of steel slag mainly includes road and mine landfill, building materials, *etc.* Among them, building materials are an effective way to improve steel slag utilization rate.^5^ Since steel slag contains dicalcium silicate (C_2_S), tricalcium silicate (C_3_S) and tetra calcium aluminoferrite (C_4_AF), steel slag has a cement-like hydration process and is considered a potential cementitious material.^6^ Using SS instead of cement to prepare green cement-based materials can improve its utilization rate, and the use of SS can reduce the amount of cement and carbon dioxide emissions.^7^ Costa *et al.* discovered that every ton of clinker saved could reduce 0.83 tons of carbon dioxide emissions and save 3.7 GJ of energy.^8^ Dong *et al.*^9^ discovered that using steel slag as aggregate to fabricate concrete could meet engineering requirements to some extent, However, the expansion caused by f-CaO and f-MgO in steel slag limits its engineering application. Fortunately, cement prepared with steel slag has better wear resistance, and a reasonable proportion can help reduce carbon dioxide emissions.^10,11^ Numerous studies have shown that,^12,13^ when the SS content is lower than 20%, SS concrete exhibits lower early compressive strength in comparation to the reference group. However, as further increasing SS content, the concrete compressive strength decreases significantly. Altun *et al.*^14^ found that using steel slag to replace 15% to 45% of cement reduced the compressive strength of cement slurry by 57.3% to 24.3% and 19.1% to 45.5% at 2 days and 7 days, respectively. Similar results were also obtained by Kourounis *et al.*,^15^ the 7 days compressive strengths of cement slurries containing 15%, 30% and 45% SS decreased by 18%, 37% and 54%, respectively, compared to the control sample. The mechanism of SS preventing early hydration of cement has been thoroughly investigated by Zhuang *et al.*^16^ The outcomes demonstrated that SS inhibition prevented C–S–H nucleation and development as well as the precipitation of Ca(OH)_2_(CH), and the SSP contains RO phase (CaO–FeO–MnO–MgO solid solution) with almost no hydration activity. The weaker interface transition zone (ITZ) formed between the RO phase and surrounding C–S–H gel, which also severely restricts the application of SS in cement-based materials.

Recent applications of nanomaterials in the field of civil engineering^17,18^ include carbon nanotubes, nanosilica, graphene oxide (GO),^19^ and carbon nanofibers, among which GO is chosen because of the extremely high specific surface area, superb mechanical strength, and amazing flexibility.^20,21^ Active surface oxygenic functional groups, including as hydroxyl and carboxyl groups in GO increase the interlamellar distance of GO, hence enhancing its dispersion in water.^22^ Numerous researchers^23,24^ have demonstrated that theses surface oxygenic functional groups of GO will preferentially react with C_3_S, C_2_S, and C_3_A. Therefore, these groups will provide growth sites for cement hydration products, which will in turn promote cement hydration. Lu *et al.*^25^ discovered that the cement-based materials with 0.08% GO had 37.7%, 24.8%, and 80.6% higher tensile strength, compressive strength, and flexural strength. Peng *et al.*^26^ confirmed that the addition of GO raised the compressive and flexural strength of fly ash cement mortar, especially the early strength. It is concluded that GO provides a new insight to overcome the shortcoming of the steel slag cement low strength in the early period.

This research evaluated the impacts of GO on the hydration and mechanical characteristics of steel slag cement utilizing XRD, SEM/EDS, and MIP tests to improve the usage of SS as a supplemental cementing ingredient in cement-based substances. Furthermore, the impacts of GO on the composition, shape, and nanomechanical characteristics of steel slag cement hydration products were examined. The mechanism of GO in steel slag cement was then investigated, which might provide a theoretical foundation for the effect of GO on steel slag cement and the application of cement-based materials.

## 2 Experimental

### 2.1 Materials

**Fig. 1:**
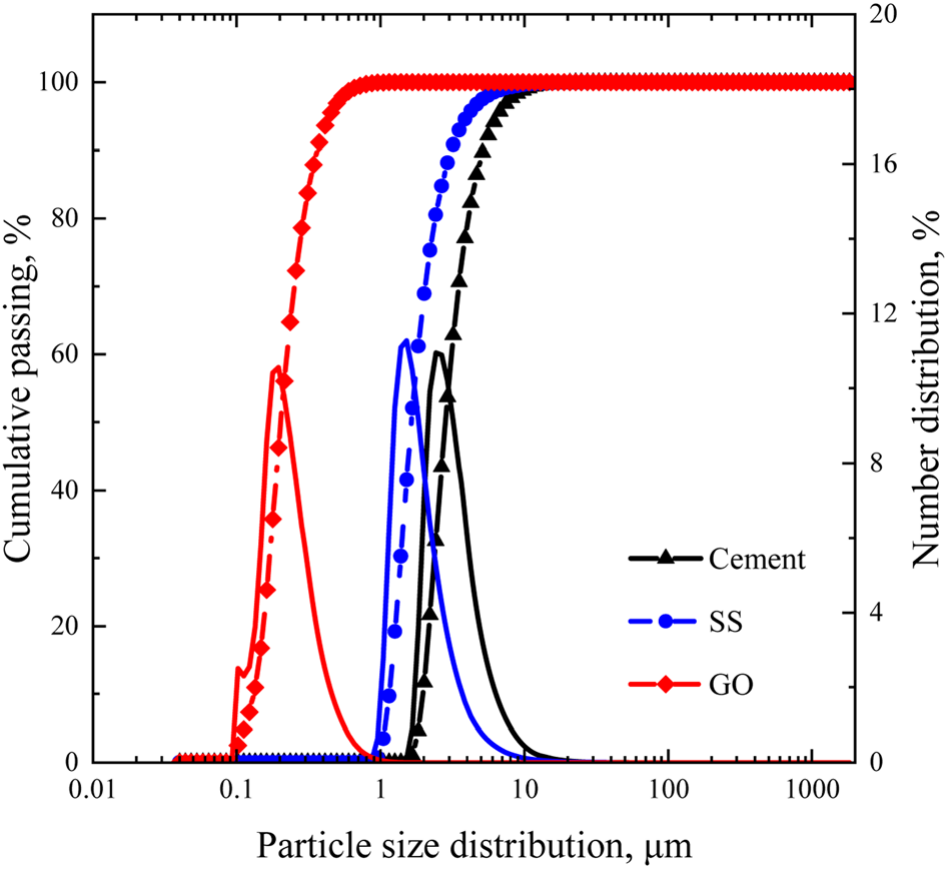
Distributions of particle size for cement, SS, and GO.

**Fig. 2:**
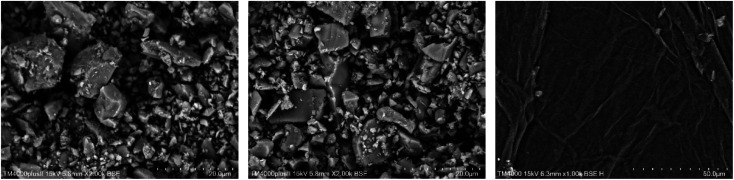
SEM images of type cement, SS and GO.

**Fig. 3:**
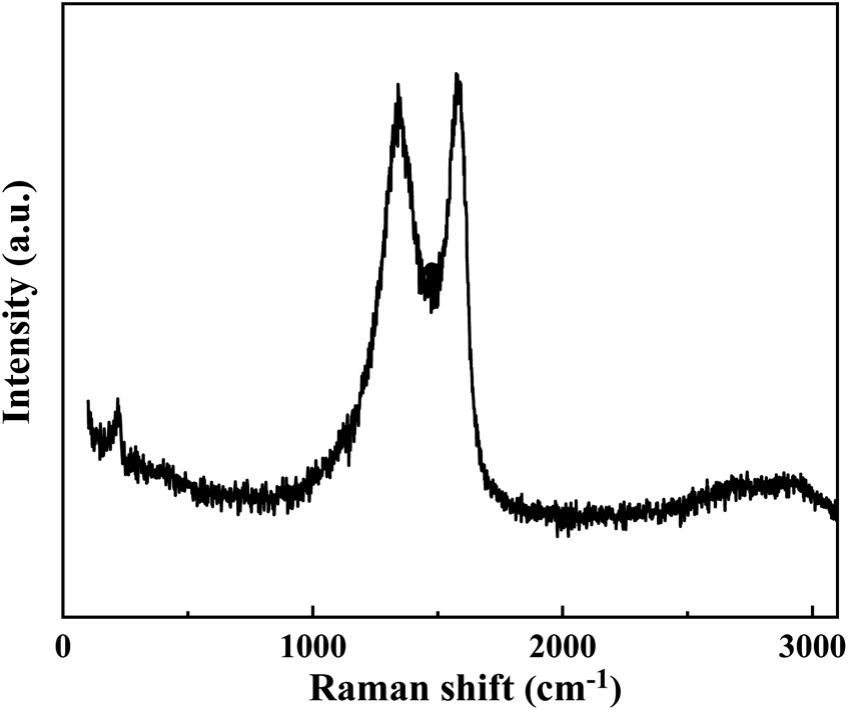
Raman image of GO.

The cement applied is P.O 42.5 Portland cement, and the SS purchased from Hengduan New Material Co., Ltd. GO dispersion was provided by Shenzhen Suiheng Graphene Technology Co., Ltd. Table 1 exhibits the chemical composition of cement and SS. The particle size distributions of cement, SS, and GO are exhibited in Fig. 1. It is evident in Fig. 2 that the morphology of SS is irregular, and the morphology of GO is sheet-like folds. Fig. 3 shows the Raman spectrum of GO. The admixture is a polycarboxylate high-efficiency water reducer produced by Subote New Material Co., Ltd. (Jiangsu, China), and the water reduction rate is 20%. Tap water is used as mixing water.

**Table 1:** Chemical compositions (by mass) of raw materials

Sample	CaO	SiO_2_	Al_2_O_3_	SO_3_	Fe_2_O_3_	MgO	NaO	K_2_O	L.O.I
Cement	62.1	20.92	4.84	2.54	3.12	0.65	0.1	0.53	3.12
SS	39.62	7.12	1.65	1.08	22.9	6.6	0.21	0.15	2.24

### 2.2 Mixing design

In this paper, four types of mix ratios were prepared to study the impact of GO on steel slag cement properties, and kept the water/binder ratio at 0.4. The pure cement system was used as the control group. SS cement is made by mixing 20% SS and 80% cement. The content of GO is 0%, 0.02% and 0.04% of the mass of steel slag cement, respectively, which are labeled as 20SS + 0GO, 20SS + 0.02GO and 20SS + 0.04GO. The mixing ratio of cement paste is demonstrated in Table 2.

**Table 2:** Mixing ratio of cement paste

Sample	Ingredients, %			
	Cement	SS	GO	Water
Control	100	0	0	40
20SS + 0GO	80	20	0	40
20SS + 0.02GO	80	20	0.02	40
20SS + 0.04GO	80	20	0.04	40

After mixing GO and stirring water, then sonicated for 60 min. Mixed GO solution with cement and then poured into a 40 cm × 40 cm × 40 cm cubic mold. After 24 hours of indoor curing, the mold was removed. Then, the mechanical properties and microstructure were tested after curing under standard conditions to the corresponding age.

### 2.3 Measurements

#### 2.3.1 Setting time

The cement paste setting time was analyzed by a Vicat apparatus in accordance with GB/T 1346-2011.

#### 2.3.2 LF-NMR

^1^H low-field nuclear magnetic resonance (LF-NMR) produced by Suzhou Niumai Electronic Technology Co., Ltd. is applied with the magnetic field intensity of (0.30 ± 0.05) *T*, the main frequency of the instrument of 12 MHZ, and the probe coil diameter of 10 mm. CPMG pulse sequence test was adopted, the sampling frequency SW was 400 kHz, the half echo time *τ* was 90 μs, the repeat sampling delay TW was 10 000 ms, and the number of echoes NECH was 80.

#### 2.3.3 Hydration heat

The TAM Air C80 Isothermal Calorimeter (age) produced in Setaram, France, with 8 channels, can be used to determine the hydration exothermal process and age rise of each sample simultaneously.

#### 2.3.4 Compressive strength

For the purpose of determining the compressive strength of cement paste at a loading speed of 2.4 kN s^−1^, a microcomputer-controlled pressure testing machine with the model number YAW-300/20 was utilized.

#### 2.3.5 XRD

XRD analyzes cement paste phase. Anhydrous ethanol was used to dehydrate the cement paste after 3 and 28 days. Cement paste fragments were 60 °C vacuum dried for 72 h, crushed into powder, and screened at 75 μm before testing. The scanning angle range is 10°–80°, and the scanning speed is 10° min^−1^.

#### 2.3.6 SEM-EDS

A scanning electron microscope (SEM) and an electron energy dispersive spectrometer (EDS) were used, respectively, to observe the microstructure characteristics of cement paste and its chemical composition. Following a curing period of 28 days, the sample was cut up into smaller pieces and then immersed to prevent further hydration in anhydrous ethanol. The material was vacuum dried for three days at a temperature of forty degrees Celsius before being tested. In the course of processing, the sample was sprayed with gold.

#### 2.3.7 MIP

Mercury intrusion porosimetry (MIP, AutoPore 9500) is applied to analyze the pore structure and distribution of cement paste samples, which were broken and took a block about 0.5 cm inside after curing for 28 days.

#### 2.3.8 Nanomechanical properties test

The nanomechanical performances of the matrix are measured by nanoindentation apparatus. The matrix is 4 × 25, including 100 indentation points, and the point spacing is 5 μm. More preparation and testing procedures are given in the recent study of Wang *et al.*^27^

## 3 Result

### 3.1 Fresh cement paste

#### 3.1.1 Setting time

**Fig. 4:**
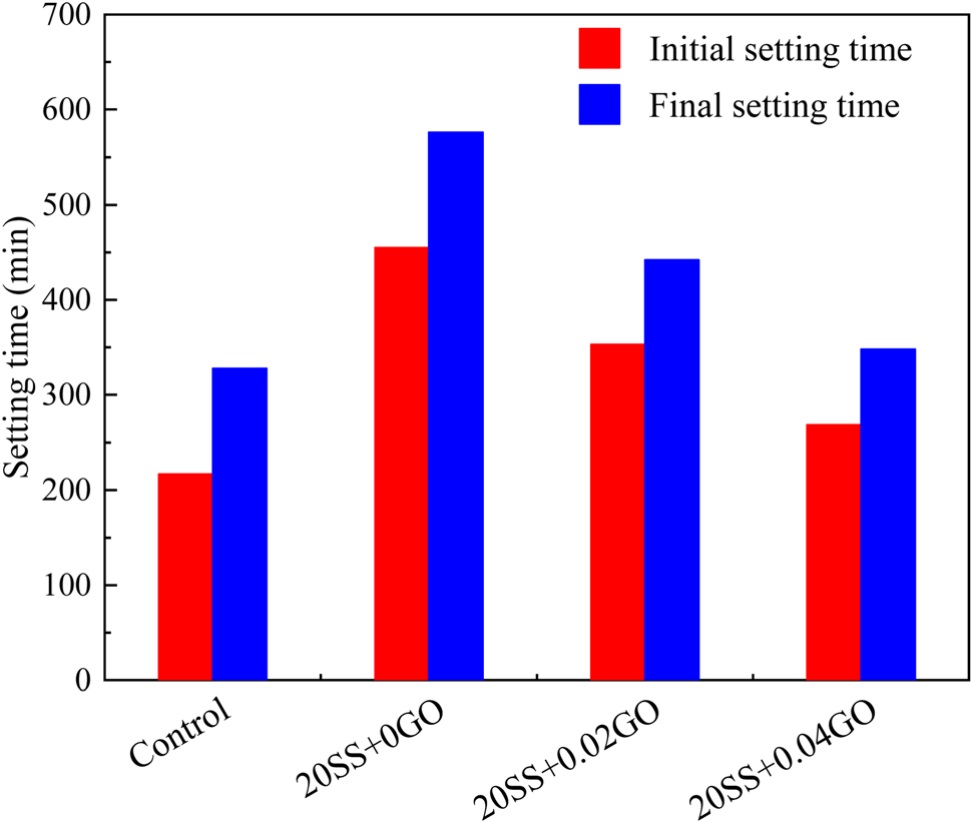
The setting time for the cement paste.

An essential sign of early age hydration is the setting time. Fig. 4 shows it clearly that the initial setting time of the sample in the reference group is 217 min, while that of the single SS doped paste is 455 min. This is consistent with the literature,^16^ where the paste containing SS initially sets at 7–9 h. But, the initial setting time of cement paste is curtailed by adding proper amount of GO into steel slag cement. Compared with 20SS + 0GO, the initial setting time of 20SS + 0.02GO and 20SS + 0.04GO samples is shortened by 102 min and 186 min, respectively. Similar trends between the final and first setting times are evident. It is found that nano – induced nucleation can be produced in the process of hydration by mixing nano – silica into cement, to produce well-developed hydration products. In contrast, the GO used in this study possesses the nano size effect due to its tiny particles, huge specific surface area, and robust hydrophilicity enabled by oxygen-containing groups, all of which serve to enhance cement particles' ability to interact with water molecules. Additionally, it can operate as a nucleation site for cement hydration product CH and induce Ca^2+^ in solution to produce C–S–H gel, all of which expedite the C_3_S hydration reaction. To shrink cement setting time, its hydration should be sped up.

#### 3.1.2 Hydration heat

**Fig. 5:**
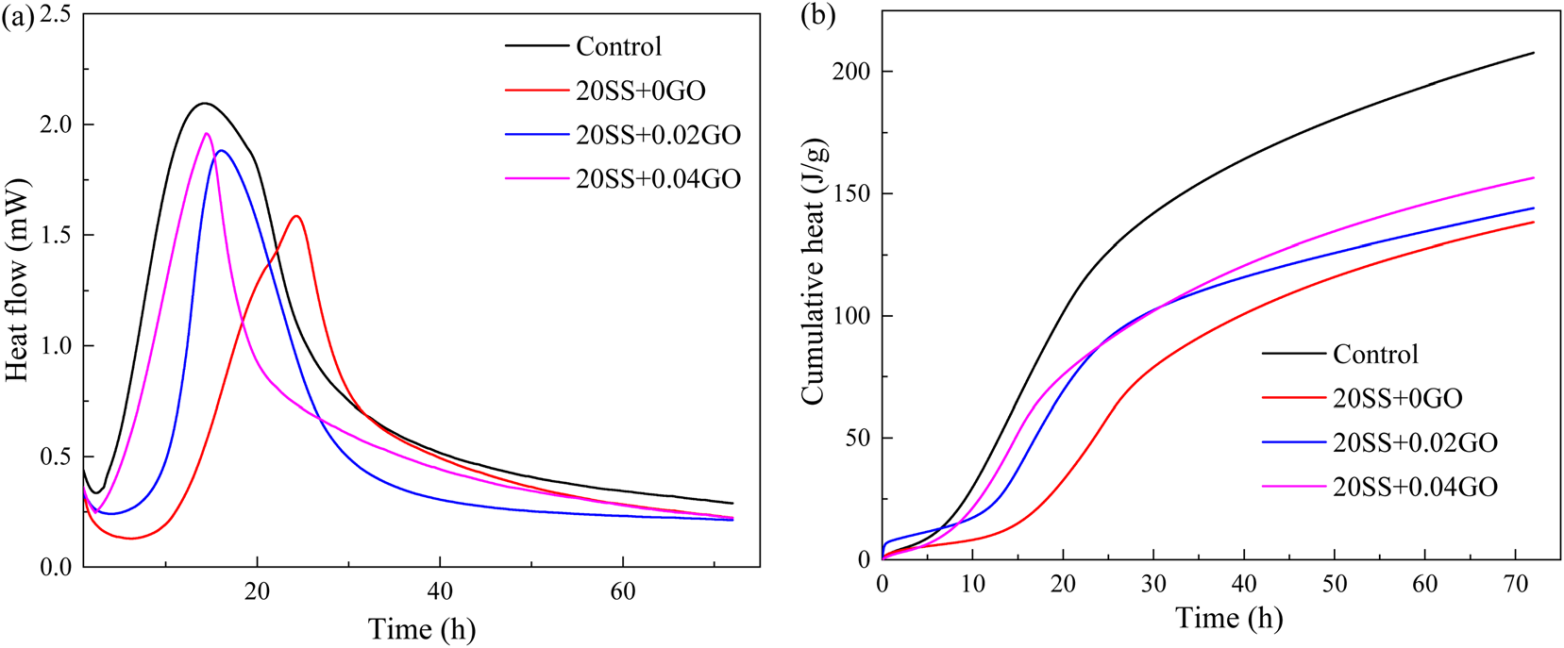
Hydration process of cement: (a) hydration heat flow, (b) cumulative heat.

To better reflect the reaction kinetics of paste, the heat release variation during the hydration of cement is evaluated by the ICC testing and the relevant results are shown in Fig. 5. It clearly shows that when SS was used instead of cement, both the largest heat flow and the total heat went down. The second exothermic peak of 20SS + 0GO had a maximum value that was 24.4% lower and a significantly delayed peak occurrence time. The cumulative heat release of the specimen for 72 h was reduced by 33.4%, in contrast to the control group. This is primarily due to the fact that the hydration heat of cement has a close relationship to its composition, the calorific value of C_3_S and C_3_A is higher, and the hydration activity of SS is significantly more sluggish than that of cement stem from partially replacement of cement by SS, reducing the amount of cement needed.^2,28^ However, with the incorporation of GO, the cement hydration process is improved. Compared with 20SS + 0GO samples, the peak heat release value of 20SS + 0.02GO and 20SS + 0.04GO samples increased by 18.7% and 23.6%, the time of peak heat release was advanced by 33.6% and 40.7%, and the cumulative heat release value of 72 h increased by 4.2% and 13.2%, respectively. Both observations of setting time are basically consistent with the ICC results.

#### 3.1.3 LF-NMR

**Fig. 6:**
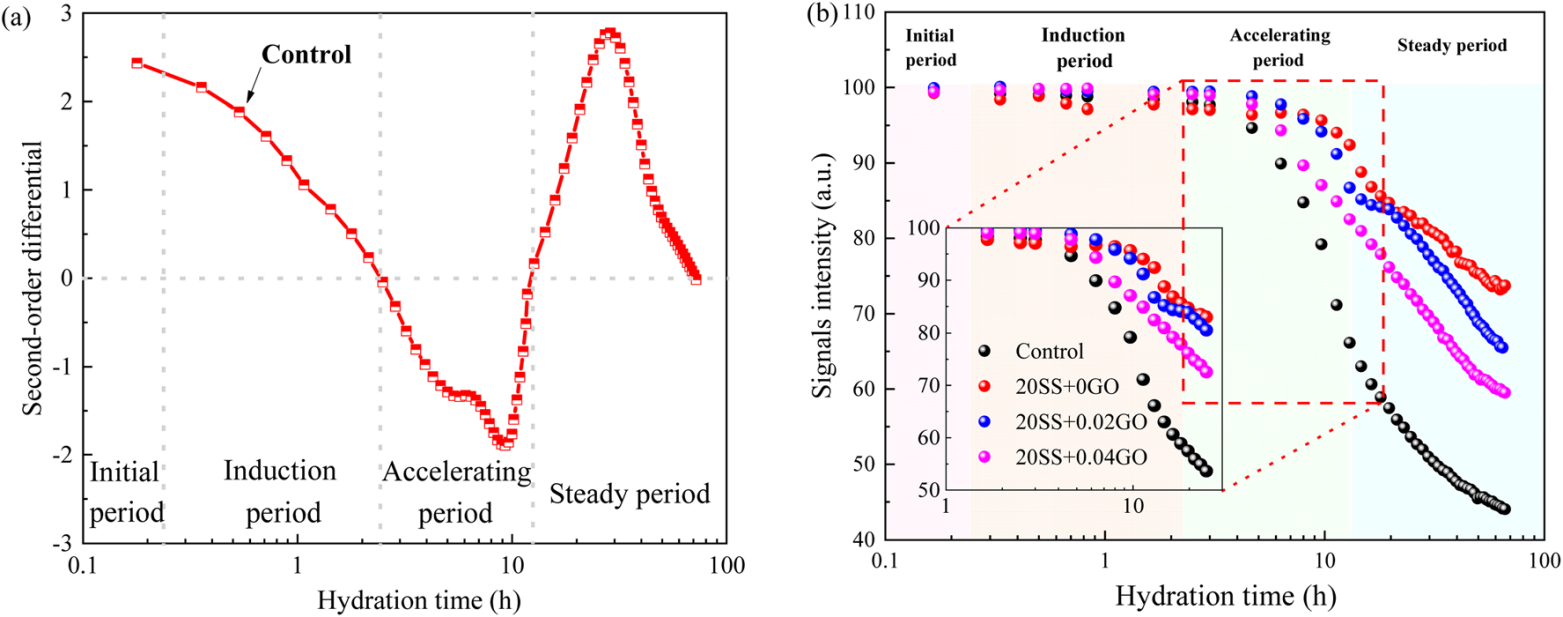
Evolution of cement paste during the hydration (a) the second-order derivative diagram of the signal intensity; (b) the total signal intensity of cement paste.

Fig. 6 depicts the evolution of cement paste during hydration. The diversification of physical bound water of cement pastes can be distinguished into four stages, in accordance with the second-order derivative diagram of the signal intensity of physical bound water of the cement paste.^29,30^ Form all curves, there is a critical time point beyond which the paste enters the accelerating stage.

Compared with control specimens, the mixing SS cement paste showed a slow decrease process, and the end time of dormant period and acceleration period were prolonged, and the transition point of the pastes shifted towards later time (Fig. 6b), implying that the hydration was prolonged by SS. This phenomenon was similar to the results of hydration heat. This is mostly owing to the poor pozzolanic reactivity of SS on the early hydration of cement, and the cement dilution effect, which causes the hydration response to be delayed. It has been widely accepted.^15,31^ However, this critical point is obviously advanced with the addition of 0.02% GO and 0.04% GO to the paste, compared to 20SS + 0GO, which correlates to the above setting time results.

### 3.2 Hardened cement paste

#### 3.2.1 Mechanical properties

**Fig. 7:**
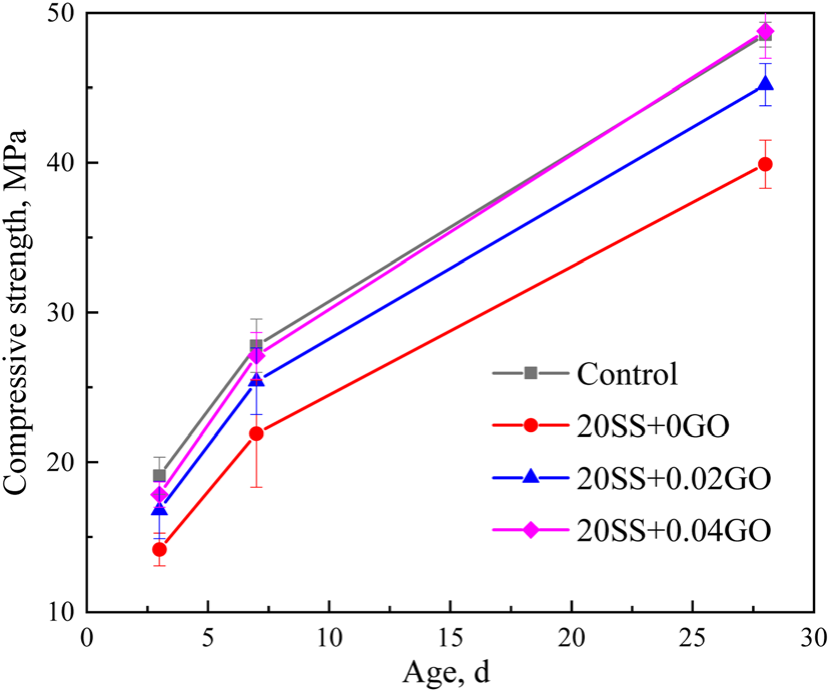
Compressive strength of slag cement paste containing GO.

**Fig. 8:**
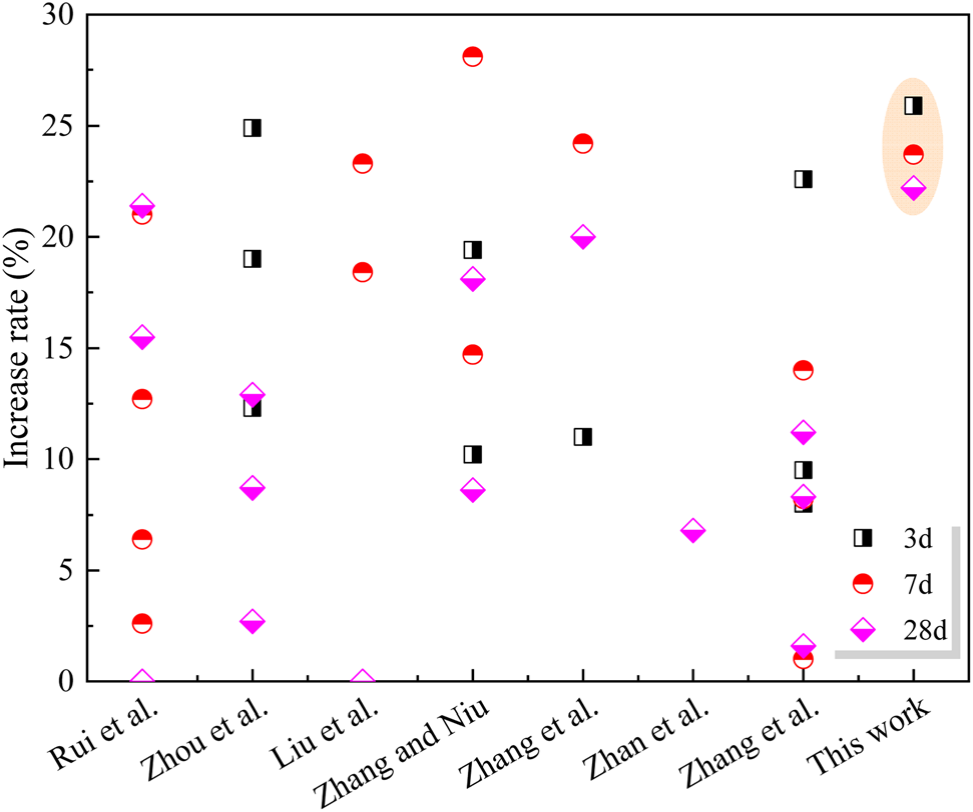
Effect of different enhancement treatment on the compressive strengths of SS-cement (Rui *et al.*,^4^ Zhou *et al.*,^34^ Liu *et al.*,^35^ Zhang and Niu,^36^ Zhang *et al.*,^37^ Zhan *et al.*,^3^ Zhang *et al.*^38^).

The cement paste compressive strength is illustrated in Fig. 7. After substituting some of the cement in the paste with SS, it was discovered that the paste compressive strength decreased over a period of 3, 7, and 28 days by a total of 25.8%, 21.1%, and 17.8%, respectively. The addition of SS leads to the reduction of cement content in the cementing system, although C_2_S and C_3_S existing in SS. Furthermore, the presence of SS at the early stage increased the Ca^2+^ concentration in pore solution, decreased the supersaturation of CH, and prevented the nucleation and development of C–S–H,^16^ resulting in a drop in strength. With increasing age, the gelling property of SS is encouraged, resulting in a slower rate of compressive strength degradation. The inclusion of GO, beyond that, increases the steel slag cement compressive strength. Compared with 20SS + 0GO, the compressive strength of the sample with 0.02% GO at 3 days, 7 days and 28 days increased by 18.6%, 15.9% and 13.3%, respectively, and that of the sample containing 0.04% GO increased by 25.9%, 23.7% and 22.2%, respectively. The 28 days compressive strength is marginally greater for the 20SS + 0.04GO sample than for the control group. It is clear that GO improves the early compressive strength of SS cement paste more significantly. This is mainly attributed to the fact that nano-scale GO fills the pores of the sample, raises the packing density, and improves the interfacial transition zone between particles.^32^ Meanwhile, GO provides additional nucleation sites for cement hydration, promoting C–S–H gel formation and cement hydration.^33^Fig. 8 summarizes the effect of different enhancement treatment on the compressive strengths of SS-cement. The combined additions of GO and 20% SSP in this study can effectively improve the compressive strength of paste, compared to other treatment me method.

#### 3.2.2 XRD

**Fig. 9:**
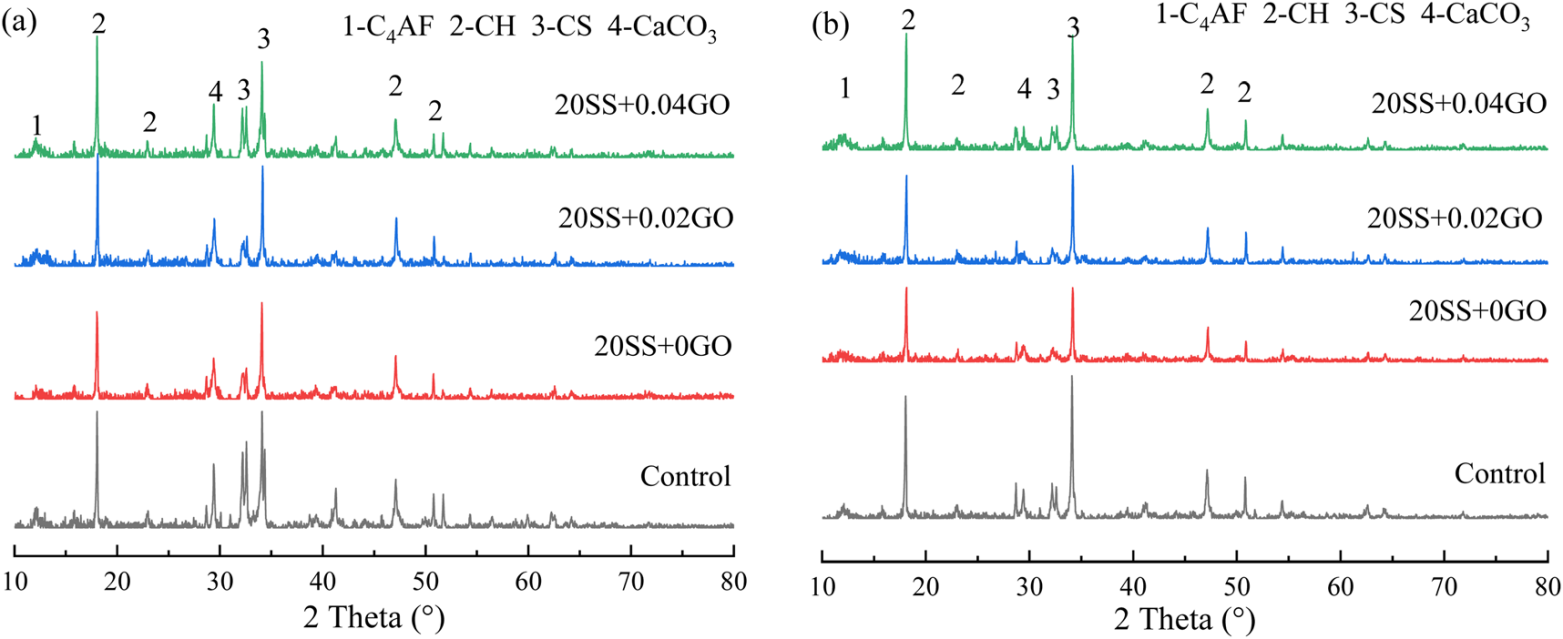
XRD patterns for the pastes at (a) 3 days and (b) 28 days.

The mineralogical compositions of the 3 days and 28 days pastes determined by XRD are presented in Fig. 9. Adding SS or GO into cement does not produce any new diffraction peaks. Unhydrated clinkers such as calcium silicate (C_3_S and β-C_2_S) and tetracalcium aluminoferrite (C_4_AF) predominate in the paste, as do CH crystals. Small amount of calcite (CaCO_3_) is also detected and it could be on account of the occurrence of carbonation. The broad peak observed in the range between 29° ∼ 30° is associated with poorly crystallized calcium silicate hydrate phase, which tends to broaden with the addition of SS. Compared with 20SS + 0GO, the incorporation of GO improves the intensity of CH crystal's diffraction peak. Most likely, this is due to the large number of hydroxyl, carboxyl and other surface oxygenic functional groups of GO, which can react with calcium ions generated by hydration, so that the content of calcium ions in hydration products decreases, and the hydration reaction of C_2_S and C_3_S moves forward.

#### 3.2.3 SEM-EDS

**Fig. 10:**
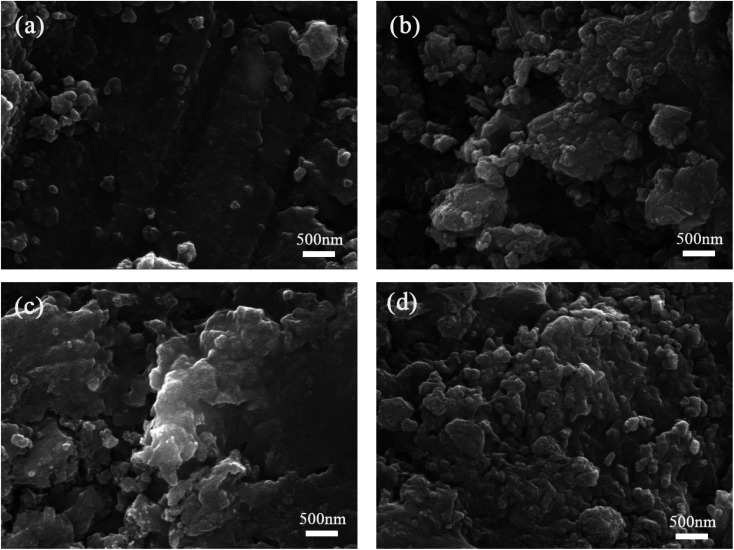
SEM photographs for cement paste at 28 days (a) control specimen; (b) 20SS + 0GO; (c) 20SS + 0.02GO; (d) 20SS + 0.04GO.

SEM photographs of the cement paste at 28 days are presented in Fig. 10. Seen from figures, adding SS weakens the cement microstructure, loosens the hydration products, and increases the holes and cracks significantly (Fig. 10b). Nonetheless, by incorporating GO, the main cement hydration products are interconnected and glomerate, forming a denser internal microstructure, and the cement paste's microstructure is improved (Fig. 10c and d). Because of small size effect, GO may fill in pores among cement paste, making the microstructure more compact. Furthermore, Ca^2+^ and Al^3+^ plasma generated by cement hydration will cross-link with –COOH on the GO surface, making hydration products to preferentially adhere to the GO surface, providing additional nucleation sites for cement hydration reaction and optimizing the crystal morphology and distribution of hydration products.^39–41^

**Fig. 11:**
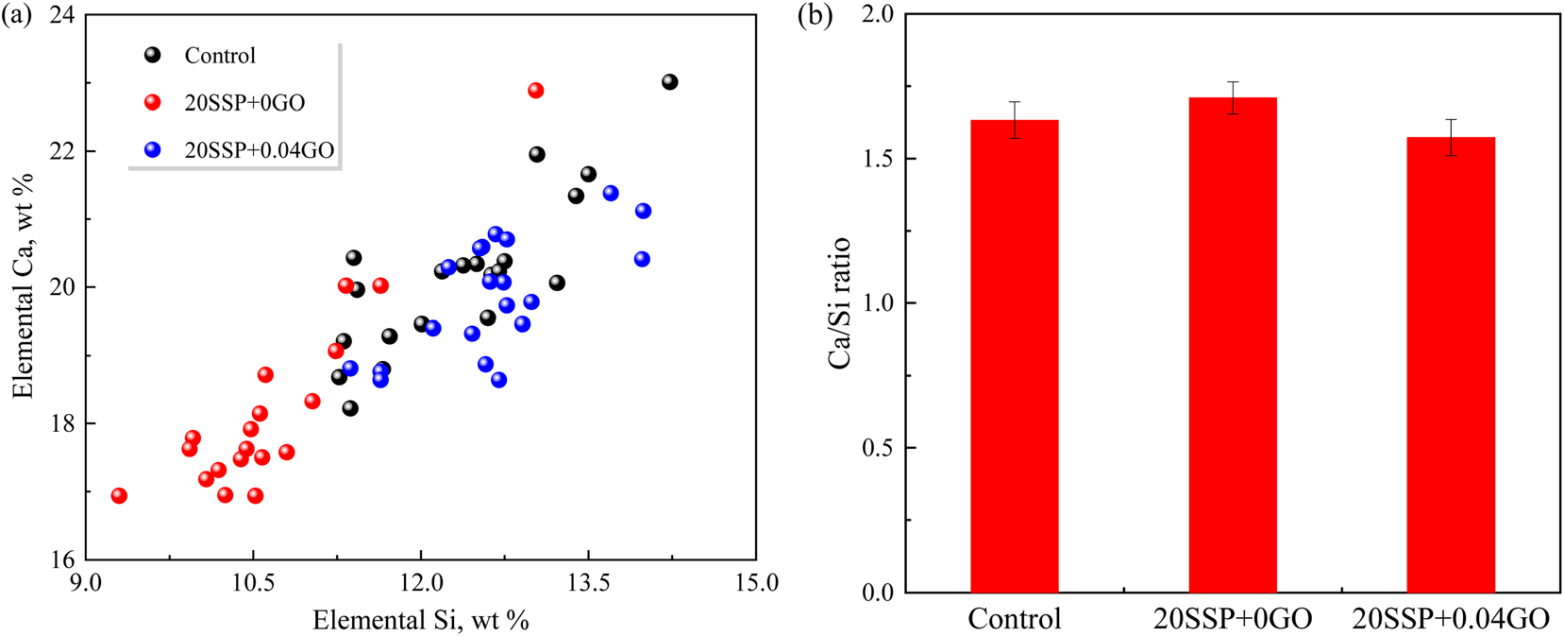
EDS results of the pastes at 28 days (a) the element content of Ca and Si; (b) Ca/Si ratio.

To explore the effect of the variation of chemical compositions on the microstructure of the pastes, the atomic ratios of the hydration products were assessed by EDS. As shown in Fig. 11, the Ca/Si ratio for the control specimen ranged from 1.45 to 1.65. The Ca/Si ratio of paste hydration products increased as SS partially replaced cement. The partial replacement of cement by SS increased the Ca/Si ratio of paste hydration products. The addition of GO, in a different circumstance, reduced the Ca/Si ratio. Earlier work confirmed that the lower Ca/Si ratio corresponded to the higher strength of paste,^42^ which was in line with our results that higher compressive strength obtained by 20SSP + 0.04GO paste.

#### 3.2.4 MIP

**Fig. 12:**
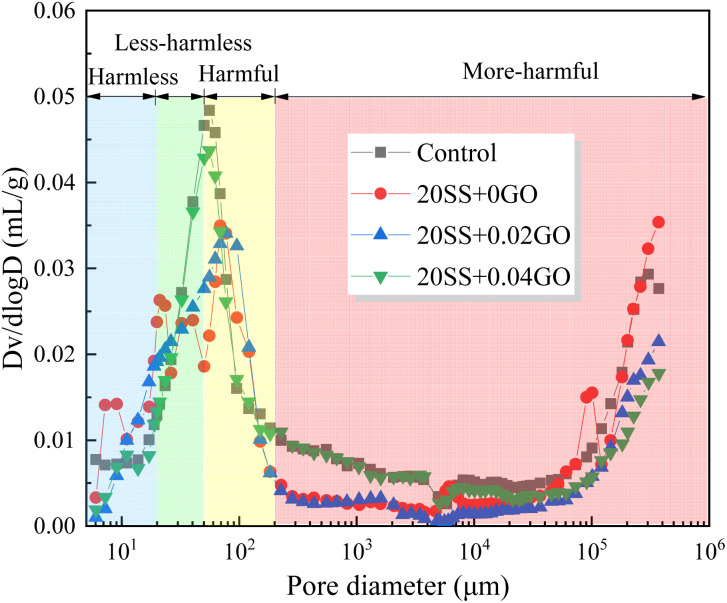
MIP curves of pastes containing SS and GO curing 28 d.

Fig. 12 depicts the evolution of the cement pore structure over the course of 28 days. According to Zhan *et al.*^43^ research, pores can be classified based on pore size and showed in Table 3. When cement is replaced by SS, the cumulative porosity of cement paste increases significantly, which is mainly attribute to the low activity of SS. However, after the addition of GO, the total porosity of the cement paste gradually decreases. Compared with 20SS + 0GO, the total porosity of 20SS + 0.02GO and 20SS + 0.04GO samples decreased by 19.1% and 33.5%, respectively. More notably, the addition of 0.02% and 0.04% GO significantly lowered the porosity of harmful pores in cement. According to the analysis, the hydration process can be accelerated by GO. Moreover, a innovative three-dimensional network architecture is established by the resulting hydration products inside the cement paste through the generation of interlacing and lapping, which filling in the cement large pores and greatly reducing the proportion of harmful pores. This also explains the 28 days mechanical property evolution law of the cement paste.

**Table 3:** Pore volume parameters of pastes

Sample	Porosity, %	Porosity in varied pore size intervals, %			
		Harmless <20 nm	Less-harmless 20 ∼ 50 nm	Harmful 50–200 nm	More-harmful >200 nm
Control	11.56	5.75	2.75	2.23	0.83
20SS + 0GO	15.2	7.60	3.39	2.45	1.76
20SS + 0.02GO	12.3	4.18	4.42	2.31	1.39
20SS + 0.04GO	10.11	4.55	2.62	2.26	0.68

#### 3.2.5 Nanomechanical properties

**Fig. 13:**
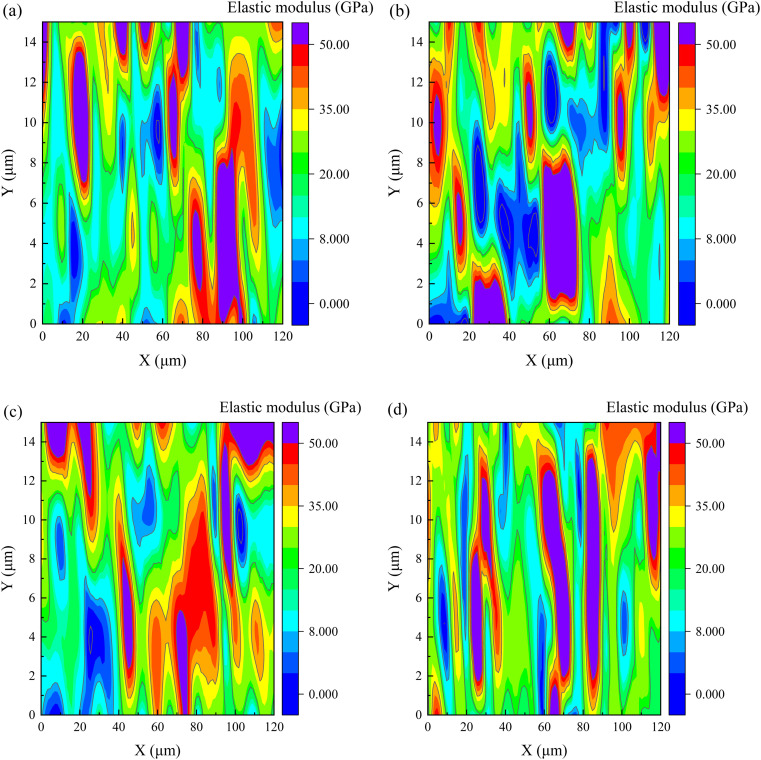
The elastic modulus at 28 days for cement matrix samples (a) control, (b) 20SS + 0GO, (c) 20SS + 0.02GO and (d) 20SS + 0.04GO.

The internal relationship between microstructure and nanomechanical properties of cement-based materials can offer theoretical basis for the study of cement-based materials. The matrix's nano-indentation elastic modulus *E* was determined using the nano-indentation test, as illustrated in Fig. 13. The elastic modulus of the matrix's distinct phases is visibly different. According to previous research results,^3,12,44^ values of the elastic modulus for cement hydration products are distributed in five regions: 0–8 GPa, 8–20 GPa, 20–35 GPa, 35–50 GPa and >50 GPa, which represent the elastic modulus of microporous, low-density (LD) C–S–H gel, high-density (HD) C–S–H gel, CH phase and unhydrated clinker (UC), respectively.

**Fig. 14:**
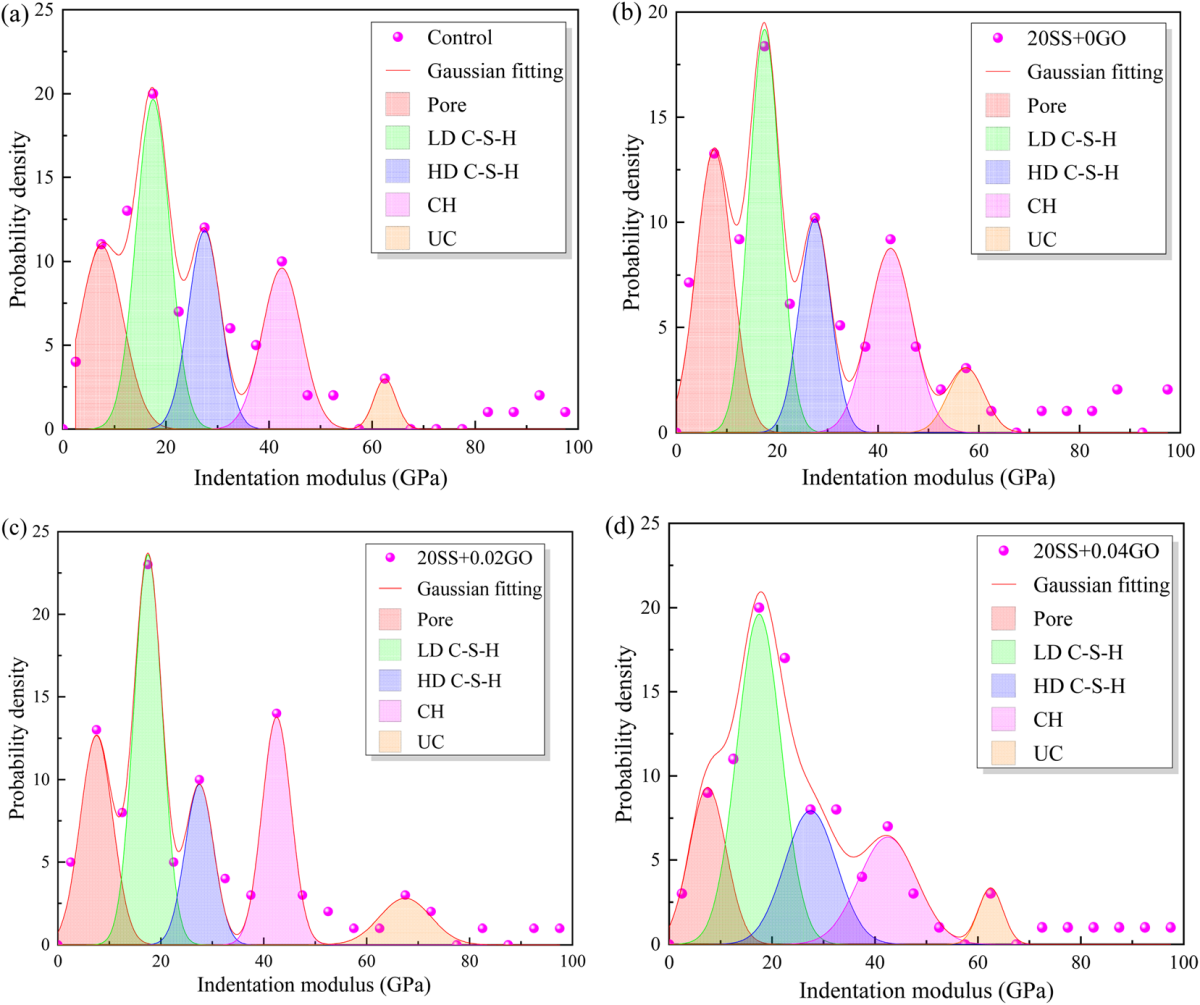
Impact of GO on the frequency distribution of cement matrix's elastic modulus (a) control, (b) 20SS + 0GO, (c) 20SS + 0.02GO, (d) 20SS + 0.04GO.

The frequency statistics of indentation modulus points in the matrix region were carried out, and the Gaussian function was adopted for curve fitting, as shown in Fig. 14. Simultaneously, through the correlation analysis of each fitting peak, the elastic modulus value and volume fraction of nano-indentation of each phase in the matrix region can be obtained, and the statistical distribution of frequency is exhibited in Table 4. When 20% SS was added, there was a clear reduction in the amount of C–S–H gel, while an enhancement in the amount of pore phase and unhydrated phase. With additional GO, however, the matrix's C–S–H gel content increased while the pore and UC phase contents decreased. A 2.2% increment in LD C–S–H content is seen in the 20SS + 0.04GO matrix compared to 20SS + 0GO, while a 55.6% increase is seen in the HD C–S–H content. In conclusion, adding enough GO can raise the C–S–H total content in the matrix, particularly the HD C–S–H. It is well known that HD C–S–H adds more strength to concrete than LD C–S–H.^45^

**Table 4:** The effect of GO on the amount of each phase in the cement matrix

Sample	Pore	LD C–S–H	HD C–S–H	CH	UC
Control	12.13	35.36	25.26	17.18	10.11
20SS + 0GO	16.33	31.64	21.43	18.37	12.25
20SS + 0.02GO	13.27	35.72	19.39	19.39	12.25
20SS + 0.04GO	11.12	32.33	33.34	14.15	9.1

## 4 Conclusions

In this study, the effect GO on the compressive strengths of SS-cement paste is investigated experimentally, and the microstructure evolution is also explained by XRD, SEM, MIP and nanoindentation techniques. The following conclusions can be drawn:

(1) SS can significantly delay the setting time of cement and reduce the hydration reaction. Compared with the control sample, adding 20% SS, the cumulative heat release of cement paste for 72 hours was reduced by 33.4%. When GO was added to SS-cement, the hydration reaction of cement was enhanced, and the peak heat release value of SS-cement paste was increased by 18.7–23.6%. LF-NMR test also obtained similar results.

(2) Compared with the control sample, the compressive strength of cement paste with 20% SS decreased by 25.8%, 21.1% and 17.7% at 3 days, 7 days and 28 days, respectively. However, with the incorporation of GO, the compressive strength of the SS-cement paste increased by 13.3–25.9%, especially in the early stage.

(3) The analysis of microscopic results shows that with the addition of 20% SS, the cement paste has a higher total porosity and a looser microstructure. However, the incorporation of GO reduced the total porosity of the SS-cement paste by 19.1–33.5%, especially the content of harmful pores. With the increase of GO content, the microstructure of the SS-cement paste was further improved, and the C–S–H gel obtained a lower Ca/Si ratio.

(4) Nanoindentation studies revealed that when 20% SS partially replaced the cement, the content of the C–S–H gel phase in the matrix decreased. With the addition of GO, the SS-cement paste obtained lower porosity, and higher C–S–H gel content. The HD C–S–H phase contents in the SS-cement matrix with the GO increased by 55.6%, further explaining the development law of the macroscopic properties of the cement-SS–GO system.

## Conflicts of interest

There are no conflicts to declare.

## Supplementary Material
